# Microarray-Based Genomic Profiling in Low-Dose Radiation Research: Evidence, Limitations, and Translational Perspectives

**DOI:** 10.3390/ijms27072942

**Published:** 2026-03-24

**Authors:** Sandugash Auganbayeva, Meruyert Massabayeva, Nailya Chaizhunussova, Dariya Shabdarbayeva, Lyudmila Pivina, Andrey Orekhov, Zhanargul Smailova, Saulesh Apbassova, Vladlena Sabitova, Tokzhan Akhmadiyeva, Saule Kozhanova, Dinara Mukanova, Murat Lepesbayev, Assel Baibussinova, Alexandra Lipikhina, Yulia Brait, Altay Dyussupov

**Affiliations:** 1 Department of Public Health, Semey Medical University, Semey 071407, Kazakhstan; sandugash_ahmetzhanova@mail.ru (S.A.); n.nailya@mail.ru (N.C.); dariya_kz@bk.ru (D.S.); lyudmila.pivina@smu.edu.kz (L.P.); andrey.orekhov@smu.edu.kz (A.O.); saulesh.apbassova@smu.edu.kz (S.A.); sabitovavladlena@gmail.com (V.S.); tokzhan.akhmadiyeva@smu.edu.kz (T.A.); apbasova65@mail.ru (S.K.); dinara.mukanova@smu.edu.kz (D.M.); king87ml@gmail.com (M.L.); assel.baibussinova@smu.edu.kz (A.B.); altay.dyusupov@smu.edu.kz (A.D.); 2 Scientific Research Institute of Radiation Medicine and Ecology, Semey Medical University, Semey 071407, Kazakhstan; a.v.lipikhina@mail.ru (A.L.); d.yuliay@mail.ru (Y.B.)

**Keywords:** low-dose ionizing radiation, DNA microarrays, transcriptomics, epigenomics, radiation biodosimetry, population studies, machine learning, reproducibility, translational radiation research

## Abstract

Low-dose ionizing radiation exposure remains a major challenge for long-term health risk assessment, particularly in retrospective cohorts with heterogeneous exposure scenarios and limited biological material. Although next-generation sequencing (NGS) technologies dominate contemporary molecular research, DNA microarrays remain relevant in radiation biology due to their standardization, reproducibility, cost-effectiveness, and compatibility with archived biospecimens. This narrative review examines the contribution of microarray-based transcriptomic and epigenomic profiling to the study of low-dose radiation effects (≤100 mSv, millisievert), with emphasis on human observational studies, radiation epidemiology, and biodosimetric applications. The literature was identified through targeted searches in PubMed and Web of Science (2000–2025). Evidence from experimental models and exposed populations is synthesized to identify recurrent molecular pathways, major sources of variability, and challenges affecting reproducibility and cross-cohort comparability. Based on this evidence, a conceptual framework is proposed to define conditions under which microarray-based analyses remain interpretable and translationally informative. Machine learning approaches are discussed in a supportive role, with emphasis on interpretability and biological plausibility. Overall, DNA microarrays are positioned as a mature, niche technology that complements next-generation sequencing platforms and remains particularly suited for retrospective cohort studies and long-term molecular monitoring in radiation research.

## 1. Introduction

Over recent decades, the biological effects of low-dose ionizing radiation have gained increasing attention due to the widespread use of radiation sources in medicine, industry, aviation, and nuclear energy [1,2]. In contrast to high-dose exposure, the biological effects of low-dose radiation are less predictable and often manifest at a subclinical level. These effects may include persistent alterations in gene expression, disruption of DNA repair mechanisms, epigenetic modifications, and the accumulation of genomic instability [3,4,5,6]. This complexity underscores the need for sensitive molecular tools capable of detecting and monitoring such changes, particularly in the context of long-term or chronic radiation exposure [3,5].

One such tool is DNA microarray technology, which enables the parallel analysis of thousands of genes, genotyping of single-nucleotide variations, detection of copy number variations (CNVs), and assessment of epigenetic alterations [7,8,9]. Owing to their high throughput, standardized platforms, and extensive history of application, microarrays have been widely used in radiation genomics, particularly for investigating individual- and population-level responses to low-dose radiation exposure [3,10].

Despite the rapid development of next-generation sequencing (NGS) technologies, DNA microarrays continue to play an important role as an accessible, validated, and interpretable approach in studies where comparative gene expression analysis, identification of radiosensitivity signatures, or large-scale monitoring of genomic damage is required [11,12]. This is especially relevant for research involving exposed cohorts, modeling of prolonged radiation exposure, and the investigation of biological effects associated with low-dose medical imaging or radiotherapy [1,2].

The aim of this narrative review is to synthesize current evidence on the application of DNA microarrays in the study of biological effects induced by low-dose ionizing radiation, with particular emphasis on interpretability, reproducibility, and translational relevance.

Importantly, this review does not aim to directly compare the intrinsic radiotoxicity of different radionuclides. Instead, it examines how microarray-based transcriptomic studies have been applied across diverse biological systems to investigate molecular responses to low-dose ionizing radiation. In addition to summarizing existing literature, this review proposes a conceptual interpretability-oriented framework designed to clarify the conditions under which microarray-based analyses remain methodologically justified and translationally informative in low-dose radiation research. Given the pronounced heterogeneity of study designs, exposure scenarios, biological materials, and analytical pipelines, conventional quantitative synthesis or meta-analysis is often inappropriate and potentially misleading in this field.

To address this challenge, we propose an interpretability-oriented analytical framework and a practical checklist designed to guide the evaluation of microarray findings in low-dose radiation studies. This framework is explicitly informed by the strengths and limitations identified through the reviewed literature and aims to support biological plausibility and translational readiness, particularly in retrospective human cohorts and archival biospecimen studies.

## 2. Search Strategy and Literature Analysis

This work is a narrative review aimed at a conceptual analysis of the application of microarray technologies in the study of biological effects induced by low-dose ionizing radiation. The review is based on a targeted and iterative examination of the scientific literature, conducted to identify key methodological approaches, characteristic transcriptomic patterns, and interpretative limitations inherent to this research field.

The literature search was performed primarily using the PubMed and Web of Science databases and covered publications released between 2000 and 2025. Search queries included combinations of terms reflecting both the radiation context and the applied molecular technologies, such as “low-dose ionizing radiation,” “microarray,” “gene expression,” “transcriptomics,” and “radiation genomics,” as well as related terms referring to epigenetic and genomic microarray-based approaches. Additional relevant publications were identified through manual screening of reference lists from key review articles and original studies.

Studies of various designs were considered, including human observational studies as a primary focus, as well as experimental investigations (in vitro systems and animal models) that contributed to understanding transcriptomic responses to low-dose radiation.

Only studies reporting original microarray-based transcriptomic, epigenomic or genomic profiling data, or providing methodologically relevant evaluations of microarray performance in low-dose radiation research, were prioritized. The selection of literature was not guided by formal criteria typical of systematic reviews but relied on expert judgment regarding the relevance, interpretability, and conceptual significance of the studies for the topic under discussion. The objective was not to provide an exhaustive coverage of all published studies, but to support a critical and interpretability-oriented synthesis of representative and methodologically informative evidence.

Given the pronounced heterogeneity of dosimetric metrics, exposure scenarios, biological materials, and analytical pipelines, neither a formal systematic review nor a quantitative synthesis of data was undertaken. Accordingly, this review does not aim to provide an exhaustive coverage of all published studies or to compile a unified list of radiation-responsive genes. Instead, it focuses on an analytical discussion of the capabilities and limitations of microarray-based approaches in the context of low-dose radiation research.

Within this review, the term low-dose ionizing radiation is used as a working concept to describe dose ranges associated with weak, variable, and context-dependent molecular responses. Depending on the study design, different dosimetric metrics are employed in the literature, including effective dose (mSv) in population-based and occupational studies and absorbed dose (mGy) in experimental and biological models. This heterogeneity reflects the intrinsic complexity of low-dose radiation research and limits direct comparability of transcriptomic findings across studies, necessitating cautious interpretation of observed molecular changes.

For the purposes of this review, the term “low-dose ionizing radiation” primarily refers to exposures below approximately 100 mSv, consistent with commonly used definitions in radiation protection and epidemiological research. However, some experimental studies discussed in the literature include dose ranges extending to approximately 0.1–0.5 Gy. Such dose ranges are sometimes used in controlled experimental settings to investigate early molecular and transcriptional responses to radiation.

## 3. Biological Effects of Low-Dose Radiation and Molecular Profiling

### 3.1. Transcriptional, Epigenetic, and Genomic Effects of Low-Dose Radiation

Exposure to low doses of ionizing radiation (<100 mSv) can induce a broad spectrum of molecular alterations that do not necessarily result in immediate clinical consequences but may trigger biological cascades associated with genomic instability, epigenetic remodeling, and dysregulation of transcriptional control [3,4,5]. In contrast to high-dose exposure, where cellular responses often manifest as apoptosis, necrosis, or mitotic catastrophe [13,14], low-dose radiation tends to elicit more subtle yet persistent biological effects. These include oxidative stress, disruption of DNA repair pathways, and modulation of gene expression involved in cell cycle regulation, inflammatory processes, and immune responses [3,6].

Numerous in vitro and in vivo studies employing microarray platforms (including Affymetrix, Agilent, and Illumina BeadArray) have identified recurring transcriptional patterns in response to radiation doses ranging from 10 to 100 mSv [10,15]. Several studies and reviews have consistently highlighted a set of candidate genes frequently reported as responsive to low-dose radiation exposure, including CDKN1A, GADD45A, FDXR, DDB2, as well as SESN1 and PCNA, which are implicated in cell cycle regulation and DNA repair processes [10,16,17].

An important source of variability in transcriptomic responses to ionizing radiation is the biological heterogeneity of the systems under study. Different cell types, including peripheral blood cells, fibroblasts, epithelial cells, and various animal tissues, differ substantially in intrinsic radiosensitivity, proliferative status, DNA repair capacity, and baseline transcriptional programs. As a result, transcriptional signatures observed in response to low-dose radiation may vary considerably depending on the cellular context. Such variability partly explains the limited overlap of gene expression signatures reported across studies and underscores the need for cautious cross-system comparisons [15,18].

Another important biological factor influencing radiation-induced transcriptional responses is the cell-cycle stage of the irradiated cells. Radiosensitivity is known to vary substantially across the cell cycle, with cells in the G2/M phase generally exhibiting higher sensitivity compared with cells in G1, while quiescent cells in G0 may respond differently. Because many radiation-responsive genes are directly involved in DNA repair, checkpoint signaling, and cell-cycle regulation, differences in cell-cycle distribution between experimental systems can substantially influence the observed transcriptional profiles. This factor may contribute to the variability of gene expression signatures reported across different studies [13,19].

In addition to differentiated somatic cell systems, pluripotent stem cells represent another biologically important model for studying radiation responses. Human embryonic stem cells and induced pluripotent stem cell-derived systems have been shown to exhibit measurable transcriptional responses to low-dose radiation exposure [20]. In these systems, radiation exposure may induce loss of pluripotency and activation of differentiation pathways rather than classical stress or apoptotic responses. Including such models helps broaden the biological perspective on radiation-induced transcriptional responses and highlights the importance of cellular differentiation status.

For example, transcriptome profiling studies using both RNA sequencing and microarray-based approaches have demonstrated that low-dose radiation exposure is associated with measurable changes in the expression of multiple genes. This includes investigations of human peripheral blood samples analyzed by RNA-seq following low-dose irradiation [18,21]. The persistence of such transcriptional signatures at later time points after exposure, as well as their dependence on dose and exposure conditions, has been discussed in the context of responses to prolonged and chronic radiation exposure.

Beyond gene expression analysis, microarray technologies have been widely applied to the profiling of epigenetic effects, particularly DNA methylation changes. Studies utilizing DNA methylation microarray platforms have shown that low-dose ionizing radiation is associated with sustained alterations in the methylation status of genes involved in DNA repair, cell cycle regulation, and stress-response pathways [4,22,23]. The literature further suggests that epigenetic modifications may contribute to long-term genomic instability, including the potential heritability of certain radiation-induced methylation changes following exposure [24].

Earlier experimental investigations have also demonstrated the utility of microarray-based platforms for analyzing radiation-induced genomic instability, including the detection of copy number variations (CNVs) [25,26]. Experimental studies employing array comparative genomic hybridization (array-CGH) and SNP microarray technologies have shown that ionizing radiation can induce de novo CNVs, which are considered one of the mechanisms underlying long-term radiation effects [26,27]. These findings have contributed to current concepts of structural genomic instability, which are now being further refined through the application of next-generation sequencing technologies [4,28].

An emerging area of research involves the integration of microarray-derived data with multi-omics platforms, such as proteomics and metabolomics, as well as with machine learning algorithms. These integrative approaches are regarded as promising tools for identifying informative biomarkers of radiation exposure; however, they require rigorous validation, careful control of overfitting, and preservation of biological interpretability [29,30,31]. It should be noted that many multi-omics integration strategies and machine learning methodologies were originally developed and validated within the field of precision oncology, and their application to radiation biology is currently at the stage of methodological adaptation and conceptual transfer.

Despite substantial progress, several unresolved challenges remain. In particular, distinguishing robust radiation-associated signatures from non-specific stress responses remains difficult due to inter-tissue and inter-model variability [15,32]. In addition, reproducibility across platforms (e.g., Affymetrix versus Illumina, microarray versus RNA-seq) and analytical conditions remains limited [11,12,32].

Collectively, these molecular effects are characterized by low-amplitude gene expression changes, high variability, and strong context dependence, which substantially complicate their detection and interpretation using transcriptomic approaches. Within this context, careful evaluation of the capabilities and limitations of microarray platforms is particularly relevant for studying the molecular effects of low-dose radiation exposure, especially in population-based and cohort studies.

### 3.2. Limitations of Artificial Intelligence Approaches in the Analysis of Microarray Data

In recent years, artificial intelligence (AI) and machine learning methods have been increasingly applied to the analysis of microarray data in radiation genomics, including tasks such as exposure classification and identification of transcriptional signatures associated with radiation dose [17,33,34]. However, in many studies their role remains largely illustrative rather than truly predictive, and the analytical potential of such approaches is often overestimated [34].

A key limitation is the high risk of overfitting, arising from the combination of high dimensionality of microarray data and limited sample sizes, particularly in human studies [11,34]. Under these conditions, models may achieve high performance within a single dataset but fail to generalize to independent cohorts. Additional challenges are posed by batch effects and platform heterogeneity, which, if insufficiently controlled, may be erroneously interpreted by algorithms as biologically meaningful signals rather than technical artifacts [11,12].

Another important limitation is the restricted interpretability of many AI-based models. In numerous studies, emphasis is placed on classification metrics, while the biological plausibility of selected features and their association with established radiobiological pathways are only superficially examined or not addressed at all [35]. In the absence of independent validation and standardized analytical pipelines, the application of artificial intelligence rarely yields robust and transferable conclusions, substantially limiting its translational value in low-dose radiation research [34].

Against this background, ongoing efforts to standardize bioinformatic pipelines and to apply AI-assisted multi-omics integration approaches remain highly relevant, particularly for the validation of stable and biologically interpretable biomarkers of radiosensitivity [30].

**Figure 1:**
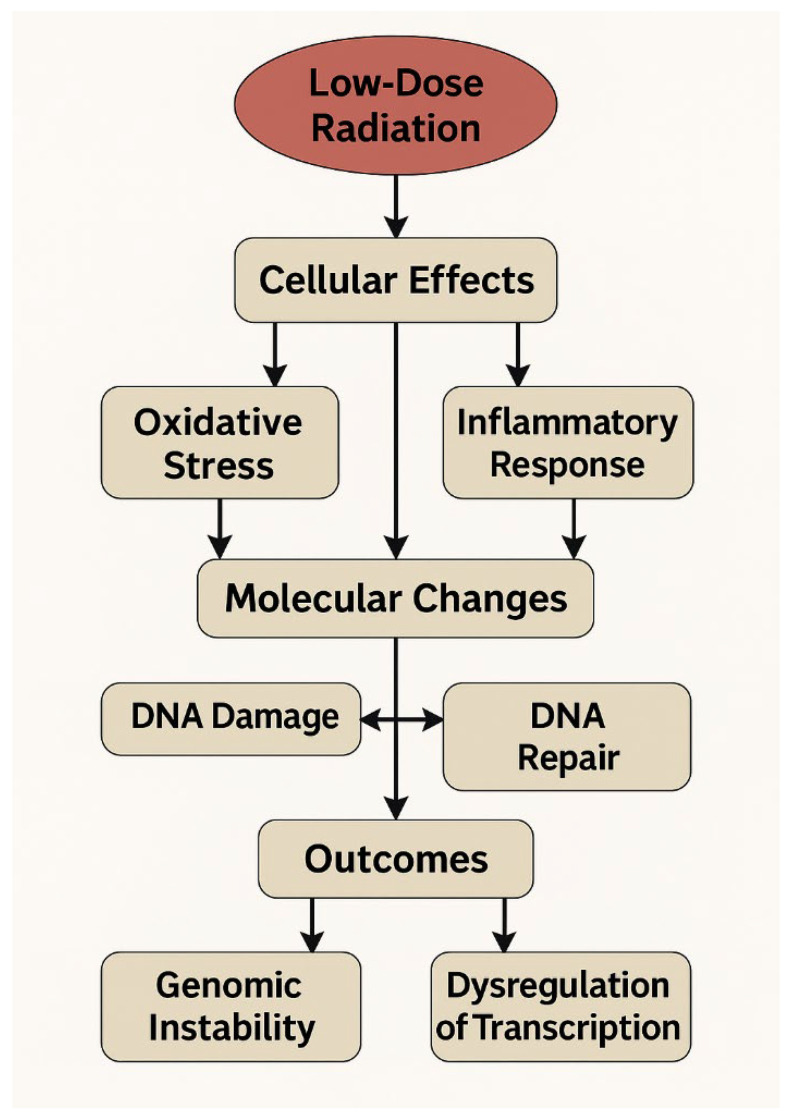
Conceptual schematic of the molecular cascade underlying cellular responses to low-dose ionizing radiation, encompassing oxidative stress, inflammatory signaling pathways, DNA damage, activation of DNA repair mechanisms, and potential long-term consequences.

A generalized model of molecular events initiated by low-dose ionizing radiation is presented in Figure 1. The schematic illustrates key stages of the cellular response, ranging from the induction of oxidative stress and inflammatory signaling cascades to the development of DNA damage, activation of DNA repair pathways, and the potential emergence of genomic instability. This cascade provides a conceptual framework for the interpretation of transcriptional signatures identified using DNA microarray technologies and facilitates the alignment of observed gene expression changes with underlying biological mechanisms of low-dose radiation exposure.

### 3.3. Preliminary Considerations for Interpretable Microarray Analysis

Based on the analysis of the published literature, DNA microarrays should not be regarded as a universal tool for identifying radiation-specific molecular signatures, but rather as a method that provides the greatest informational value under well-defined research scenarios. In particular, microarray platforms are most informative for comparative analyses of predefined gene sets, retrospective cohort studies, and long-term molecular monitoring, where repeated sample collection is not feasible and cross-cohort comparability is essential.

The application of artificial intelligence methods in the analysis of microarray data is justified only when several key conditions are met, including dimensionality reduction, rigorous control of batch effects, availability of independent datasets for validation, and mandatory biological interpretation of selected features. In the absence of these requirements, AI-based models do not enhance the reliability of conclusions and may instead amplify false reproducibility. Accordingly, the optimal strategy is not the isolated use of microarrays or machine learning algorithms, but their targeted integration within standardized analytical pipelines designed to ensure reproducibility and translational relevance of the results. These considerations form the basis for the structured interpretability framework proposed below.

## 4. Application of Microarrays in Radiation Genomics: From Models to Human Studies

**Figure 2:**
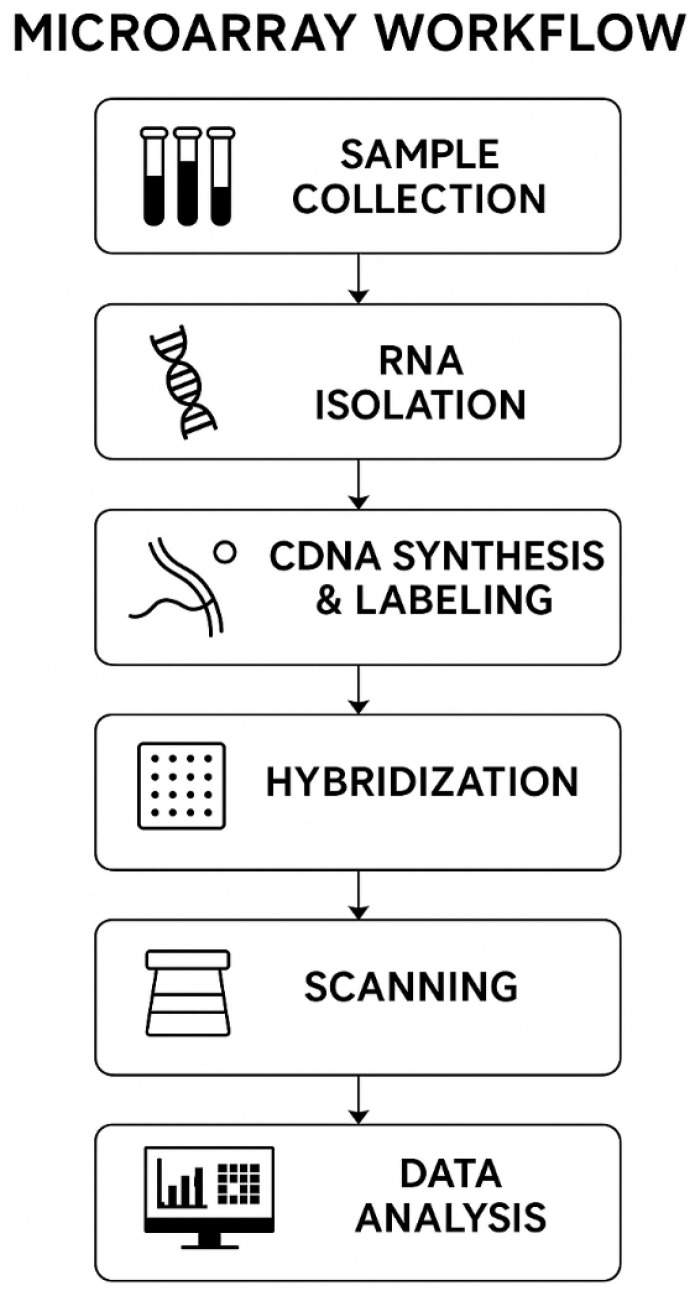
Standardized workflow of microarray analysis, including sample collection, RNA extraction, cDNA synthesis and labeling, microarray hybridization, scanning and data acquisition, and subsequent bioinformatic analysis. The schematic illustrates the main experimental stages commonly applied in radiation genomics studies.

Before addressing the application of microarray technologies in experimental models and population-based studies, it is important to outline the technical sequence of microarray analysis. Figure 2 presents a standardized workflow encompassing the key stages of sample preparation, hybridization, and subsequent bioinformatic processing. This schematic serves as a reference framework for interpreting the materials discussed in the following sections.

### 4.1. In Vitro and Preclinical Models

The investigation of molecular consequences of low-dose ionizing radiation under controlled in vitro conditions and in animal models remains a central area of radiobiological research. These systems enable detailed characterization of transcriptional and epigenetic changes at early time points after exposure, as well as assessment of dose-dependent effects that are largely unaffected by confounding factors inherent to population-based studies [18,21].

In human in vitro cellular models, including primary fibroblasts, epithelial and endothelial cells, as well as in studies of human peripheral blood, transcriptomic analyses have been widely applied to evaluate differential gene expression following low-dose ionizing radiation exposure [18,21]. Several experimental studies have demonstrated that the most consistently responsive markers of radiation exposure include genes involved in cell cycle regulation and DNA repair (e.g., CDKN1A, GADD45A, DDB2), as well as stress-response and metabolic genes such as FDXR and SESN1 [10,18].

Animal models, particularly mice and rats, allow the investigation of long-term effects of low-dose ionizing radiation across multiple target tissues, including the liver, bone marrow, and lungs. Experimental studies have shown that under chronic or fractionated exposure conditions, transcriptional alterations may persist over extended periods and involve not only DNA damage-related genes but also pathways associated with inflammation, cell proliferation, and tissue remodeling [18,22]. These findings underscore the importance of temporal and tissue-specific analyses of molecular responses to radiation exposure.

An additional advantage of preclinical models is the feasibility of integrating transcriptomic profiling with metabolomics and other omics-based approaches, enabling reconstruction of a more comprehensive molecular response of tissues to radiation under conditions modeling chronic and/or repeated exposures [30,36].

At the same time, several limitations must be acknowledged. Cellular models exhibit limited fidelity in reproducing in vivo responses, and findings obtained in laboratory animals are not always directly extrapolatable to humans. Nevertheless, preclinical models provide a valuable framework for generating reference transcriptional signatures, which can subsequently inform the interpretation of population-based and clinical studies [15,18].

### 4.2. Population-Based and Cohort Studies

The application of microarray technologies in population-based studies of low-dose radiation exposure represents one of the most methodologically challenging areas of radiation genomics. Unlike experimental model systems, where exposure conditions are tightly controlled, population cohorts are characterized by substantial heterogeneity in radiation dose, timing of exposure, lifestyle factors, and genetic background. Nevertheless, such studies are essential for evaluating the biological relevance of identified molecular signatures under conditions that more closely reflect real-world exposure scenarios.

Only a limited number of human studies have applied microarray-based transcriptomic approaches to the investigation of low-dose ionizing radiation exposure. Existing studies predominantly rely on peripheral blood samples and report subtle, pathway-level transcriptional alterations rather than robust gene-specific biomarkers, as summarized in Table 1. The studies presented in Table 1 were selected to illustrate key characteristics and methodological limitations of human microarray-based transcriptomic research in low-dose radiation settings, rather than to provide an exhaustive overview of the available literature.

Despite the limited number of primary human transcriptomic studies using microarray platforms (Table 1), the summarized examples reveal recurring methodological constraints, including limited statistical power, heterogeneity in dose assessment, and partial overlap of radiation-responsive gene sets with broader stress-related pathways. These shared limitations underscore the interpretative challenges inherent to population-based low-dose studies and provide direct justification for the structured framework proposed below.

Beyond these primary microarray-based investigations, a broader body of evidence derived from population-based and cohort studies employing diverse transcriptomic approaches including RNA sequencing and re-analysis of archived microarray datasets allows for discussion of general patterns of molecular responses to low-dose radiation exposure under real-world conditions. The most prominent publications concern cohorts of Chernobyl cleanup workers, nuclear industry employees, and populations exposed to environmental radiation [21,42,43].

Several studies employing transcriptomic profiling, predominantly based on RNA-seq as well as re-analysis of archived Affymetrix and Illumina microarray datasets, have reported alterations in the expression of genes involved in DNA damage response, cell cycle regulation, reactive oxygen species metabolism, and immune-inflammatory pathways. In some investigations, these expression patterns exhibited dose-dependent characteristics and were consistent with individually reconstructed radiation doses [21,39,44].

Particular interest has been directed toward studies of populations exposed to chronic low-dose environmental radiation, including regions adjacent to nuclear test sites and uranium mining areas [45,46,47]. Within these settings, the persistence of long-term molecular alterations in peripheral blood has been discussed, with some changes remaining detectable many years after exposure. These alterations potentially involve signaling pathways associated with regulation of inflammatory responses and cell proliferation, including the TGF-β and MAPK cascades. Such molecular patterns have been proposed as a possible basis for subclinical inflammatory processes and dysregulation of cellular growth control [20,21,48].

Despite the significance of these findings, population-based investigations face several challenges, including variability in exposure levels, confounding factors such as age, smoking status, and occupational risks, limitations in the availability of biological material, and the need for large-scale bioinformatic normalization and harmonization. In addition, cross-platform comparison of results is complicated by differences in array design and data processing algorithms.

Nevertheless, microarray technologies may remain a valuable tool for identifying transcriptomic response patterns to low-dose radiation exposure in retrospective population-based studies and biomonitoring programs, provided that strict quality control measures are applied and results are interpreted within a structured and transparent analytical framework.

### 4.3. Perspectives: Microarrays in Personalized Radiation Medicine and AI-Driven Analytics

Concepts of personalized medicine are increasingly being adopted in radiation biology, where growing attention is paid to the need to account for individual molecular responses to low-dose ionizing radiation. In this context, microarray technologies are viewed not only as tools for retrospective analysis but also as potential components of future diagnostic and prognostic platforms. Their application enables the assessment of inter-individual variability in radiosensitivity, modeling of long-term molecular risks, and the development of personalized strategies for radiation protection [49].

One promising direction involves the development of radiosensitivity panels based on stable transcriptional signatures. Such panels may include genes previously reported to be responsive to low-dose ionizing radiation (e.g., CDKN1A, FDXR, SESN1, PCNA) and may be complemented by genetic variants in key regulators of DNA repair and stress-response pathways [10,50]. Multigene models of this type have been tested in various experimental and clinical cohorts and have demonstrated potential for individual dose stratification and assessment of radiosensitivity [10,51].

In parallel, there is increasing interest in the application of artificial intelligence (AI) methods for the interpretation of microarray data. Machine learning algorithms, including random forest, support vector machines (SVM), and neural networks, have been applied to tasks such as automated classification of samples according to radiation exposure level, prediction of persistent molecular effects based on transcriptional patterns, and identification of minimal marker sets with high predictive value. Several studies have demonstrated that these approaches, particularly when combined with multi-omics data integration, show considerable potential for biomarker discovery and assessment of radiation sensitivity [52,53,54,55,56,57,58,59,60,61,62].

**Figure 3:**
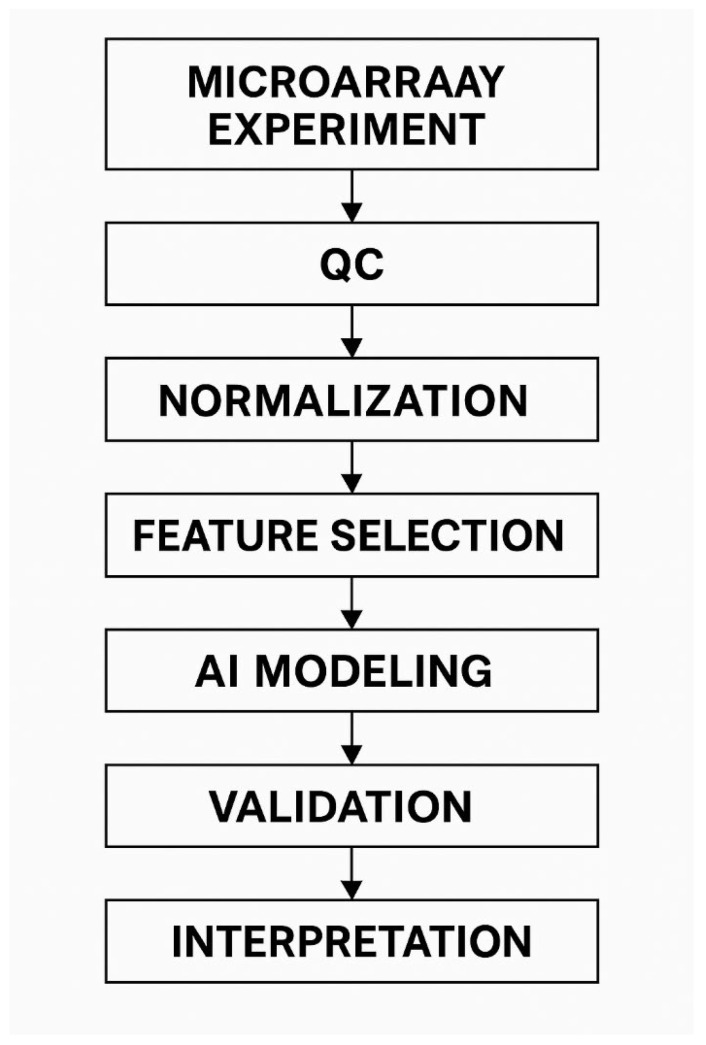
Multistep pipeline for the integration of microarray data with artificial intelligence methods, including quality control (QC), normalization, feature selection, construction of machine learning models, model validation, and biomarker interpretation. The schematic reflects contemporary approaches to the development of predictive models of radiosensitivity.

To illustrate the sequence of analytical steps required for integrating microarray data with machine learning methods, Figure 3 presents a standardized “AI + microarray” pipeline. The schematic outlines the main stages of analysis, ranging from quality control and signal normalization to feature selection, model construction, and subsequent validation.

Another emerging direction involves the development of portable microarray-based rapid testing systems (lab-on-a-chip platforms) for field-based or clinical biomonitoring applications [55]. Although such solutions are still at an early stage of development, they are already being evaluated in pilot studies, including projects involving military personnel and workers in the nuclear industry.

Overall, microarray technologies continue to retain their relevance, particularly when archived samples are used, amid the ongoing transformation of radiation biology toward a personalized, prognostic, and predictive paradigm. Their integration with multi-omics platforms and advanced analytical methods enables a transition from descriptive observation to biological interpretation and, in the longer term, to molecular data-driven decision-making.

## 5. Current Role of Microarrays in Radiation Genomics

### 5.1. General Patterns and Discrepancies in the Data

Recent studies addressing the molecular consequences of low-dose radiation exposure reveal both recurrent transcriptional patterns and substantial heterogeneity of results [28,46]. Reproducible expression changes have been reported for genes involved in DNA repair (e.g., GADD45A, CDKN1A, FDXR), inflammatory signaling cascades (IL6, TNF), cell cycle regulation (CCNG1, PCNA), and mitochondrial functions [10,19]. These findings have been observed across experimental animal models as well as in human peripheral blood samples.

At the same time, a wide range of discrepancies has been documented [48]. Such inconsistencies may arise from variability in radiation dose and exposure regimens (acute versus chronic), tissue-specific responses, differences between analytical platforms (microarrays versus RNA-seq), timing of sample collection after exposure, and biological heterogeneity of the studied cohorts, including age, sex, and underlying health conditions.

Against the backdrop of the rapid expansion of next-generation sequencing (NGS) technologies, such as RNA-seq and whole-genome sequencing (WGS), microarrays are increasingly perceived as outdated tools [19,56,57,58]. However, closer examination suggests that these platforms do not so much compete as fulfill distinct roles within an integrated genomic strategy.

### 5.2. Methodological Limitations of Current Studies

An analysis of the published literature on the molecular effects of low-dose ionizing radiation highlights several systemic methodological limitations that substantially affect data interpretation and translational applicability. Despite the considerable volume of accumulated microarray-based datasets, the reproducibility of reported transcriptional signatures remains limited, underscoring the need for cautious and critical interpretation of these findings.

### 5.3. Limited Reproducibility of Transcriptional Signatures: A Generalized Analysis of Contributing Factors

A key challenge in studies of low-dose radiation exposure is the limited specificity of transcriptional responses. Gene expression changes detected at doses below 100 mSv often overlap with universal cellular stress responses, including oxidative stress, inflammation, hypoxia, and metabolic dysregulation [30,59]. As a result, genes traditionally considered markers of radiation exposure (e.g., CDKN1A, GADD45A, FDXR) frequently reflect a generalized stress response rather than radiation-specific effects, particularly in the absence of appropriate control groups and multifactorial analytical frameworks [60,61].

An additional source of inter-study variability arises from the high sensitivity of microarray platforms to both technical and biological variation [62]. Differences in array design, normalization procedures, and statistical filtering strategies reduce the comparability of results across platforms and cohorts, especially for genes expressed at moderate or low levels [63]. These effects are further exacerbated by small sample sizes, which are characteristic of most human studies, increasing the risk of false-positive findings and diminishing the stability of identified signatures upon independent validation [64].

Temporal instability of transcriptional responses represents another major limitation. A substantial proportion of gene expression changes induced by low-dose radiation exposure are transient and highly dependent on the timing of sample collection [18,65]. The lack of standardized temporal study designs complicates the identification of persistent long-term molecular effects and further undermines reproducibility across studies [41,66].

Finally, conceptual limitations inherent to microarray platforms must be considered. Their reliance on fixed probe designs targeting predefined transcripts precludes the detection of novel isoforms, alternative splicing events, and non-coding RNAs, which are increasingly recognized as important contributors to cellular responses to low-dose radiation exposure [58,67,68]. Consequently, a portion of biologically relevant information remains inaccessible to microarray-based analyses.

Taken together, these factors help explain why a substantial proportion of published transcriptional signatures associated with low-dose radiation exposure fail independent validation and cannot be readily translated into clinical or population-level applications. This underscores the need to move beyond purely exploratory approaches toward standardized analytical strategies focused on reproducibility, biological interpretability, and independent confirmation of results [66,69].

### 5.4. Summary of Key Methodological Limitations

In summary, the major methodological limitations of microarray-based studies investigating the effects of low-dose radiation exposure include:

(i) Limited reproducibility of transcriptional signatures across different platforms;
(ii) Small sample sizes and insufficient statistical power;
(iii) Lack of standardized analytical pipelines;
(iv) Limited independent validation of identified markers;
(v) Insufficient consideration of the temporal dynamics of molecular changes following exposure.

### 5.5. Relevance of Microarray Platforms in Retrospective Studies

Against the backdrop of the rapid expansion of next-generation sequencing (NGS) technologies, such as RNA sequencing and whole-genome sequencing (WGS), microarrays are increasingly regarded as outdated tools [57,58,62]. However, closer examination indicates that these platforms do not primarily compete with each other but rather serve distinct purposes within an integrated genomic strategy (Table 2).

Microarray technologies are associated with several well-recognized limitations, including the inability to detect novel transcripts, a restricted dynamic range, and a fixed array design that predetermines the targets of analysis [57,58]. Nevertheless, these limitations are offset by a high degree of standardization, rapid data generation, and the availability of extensive archived datasets. This is particularly relevant in radiation biology, where repeated sample collection is often not feasible. In contrast to NGS-based approaches, microarrays-when applied using standardized analytical workflows-tend to demonstrate greater robustness in cross-cohort comparisons and remain an remain useful under defined conditions for screening predefined gene panels [62,70].

It should be emphasized that the comparison between microarray and sequencing-based technologies presented in Table 2 is generalized and context-dependent. Parameters such as reproducibility, sensitivity, and suitability for downstream analyses are strongly influenced by study design, sample quality, preprocessing methods, normalization strategies, and batch-effect correction. This consideration applies both to inter-platform comparisons of microarray data and to the comparability of RNA-seq results across different studies and analytical pipelines.

It is precisely in the context of retrospective molecular analyses, dose stratification, and long-term follow-up of exposed cohorts that microarrays represent a unique and still highly relevant resource. Accumulated microarray datasets may be viewed as the “archival fabric” of radiation genomics, which can be “reactivated” using contemporary bioinformatic approaches. Re-annotation, cross-platform normalization, and integration with AI-based models enable the development of robust radiosensitivity panels that apply to population-based biomonitoring and personalized risk assessment [30,54,60].

In the context of long-term human exposure to low-dose ionizing radiation, microarray technologies remain particularly useful for population-based studies and retrospective cohort analyses. Compared with next-generation sequencing approaches, microarrays offer several practical advantages in such settings, including lower cost, standardized analytical pipelines, and compatibility with archived biospecimens.

These characteristics make microarrays especially suitable for large-scale epidemiological studies and biomonitoring programs in populations exposed to chronic or environmental radiation, where repeated sampling and deep sequencing may not be feasible. Although RNA-sequencing provides higher sensitivity and broader transcriptome coverage, microarrays remain a practical and efficient option for comparative gene expression analysis, validation of predefined gene expression panels, and cross-cohort studies focusing on long-term molecular effects of radiation exposure.

Accordingly, rather than framing microarrays and NGS technologies as competitors, it is more appropriate to consider them as complementary tools, with microarrays serving as a bridge between the past and the future of molecular radiation diagnostics.

## 6. Conceptual Framework for the Application of DNA Microarray Technologies in Low-Dose Ionizing Radiation Research (Figure 4)

Across the experimental and population-based studies reviewed above, reported transcriptomic responses to low-dose ionizing radiation demonstrate substantial heterogeneity in both magnitude and direction.

This variability extends beyond biological differences and recurrently reflects inconsistencies in exposure characterization, biospecimen selection and timing, microarray platform choice, preprocessing pipelines, statistical modeling, and validation strategies.

As demonstrated throughout the preceding sections, failure to explicitly account for these contextual factors complicates cross-study comparison and increases the risk of overinterpretation of weak or context-dependent gene expression signals.

**Figure 4:**
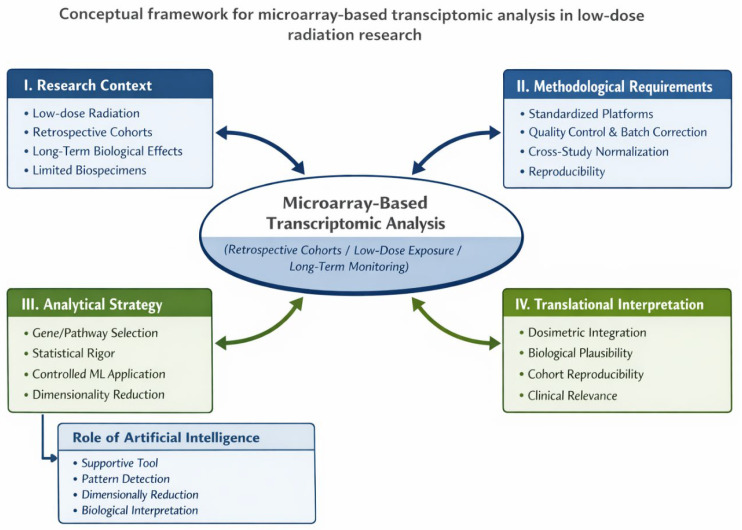
Conceptual framework for the interpretable application of DNA microarray technologies in low-dose ionizing radiation research. The framework was developed by the authors based on critical synthesis of the reviewed literature.

To address these recurring challenges identified through critical appraisal of the reviewed literature, we developed an interpretability-oriented analytical framework that systematizes key methodological, biological, and translational factors influencing the robustness of microarray-based low-dose radiation studies.

The proposed framework defines conditions under which microarray-based transcriptomic analyses remain methodologically justified and translationally informative in studies of low-dose radiation exposure. It integrates four interconnected components: (I) research context, (II) methodological requirements, (III) analytical strategy, and (IV) translational interpretation. Within this framework, DNA microarrays are positioned as a mature, niche technology complementary to next-generation sequencing platforms, particularly suited for retrospective cohort studies and long-term molecular monitoring, where reproducibility, interpretability, and cross-cohort comparability are critical. Artificial intelligence methods are incorporated in a supportive role to enhance pattern detection and dimensionality reduction while preserving biological interpretability and minimizing the risk of overfitting.

### Operationalization of the Interpretability Framework

To enhance practical applicability, the proposed framework can be translated into a structured checklist that summarizes key domains determining the interpretability and reproducibility of microarray-based studies of low-dose radiation. Each domain reflects recurrent sources of methodological and biological variability identified across the reviewed literature and highlights common limitations that may compromise meaningful biological inference or translational interpretation.

To facilitate transparent and reproducible application of the conceptual framework, its key components were translated into a structured interpretability and reproducibility checklist.

Table 3 summarizes the key interpretability domains derived from the proposed framework and translates them into practical evaluation criteria applicable to microarray-based studies of low-dose radiation exposure. For each domain, representative methodological requirements are contrasted with commonly observed limitations, providing a structured basis for assessing study robustness and for contextualizing heterogeneity across published findings.

Importantly, failure to satisfy individual domains does not necessarily invalidate a study; however, cumulative weaknesses across multiple domains substantially limit interpretability, particularly in the low-dose context where biological signal-to-noise ratios are inherently low. The framework therefore facilitates structured comparison across studies and provides a coherent basis for explaining divergent transcriptomic findings reported at comparable dose ranges.

The proposed framework is particularly relevant for machine learning-based analyses of microarray data. In the absence of robust exposure characterization, preprocessing transparency, and biological validation, machine learning models are prone to capturing cohort-specific or technical artefacts rather than radiation-associated signals. Consequently, application of advanced analytical approaches should be contingent upon satisfying minimum interpretability criteria across the outlined domains.

While the framework has not been formally validated, it is grounded in recurrent methodological patterns observed across independent studies.

## 7. Limitations of the Review

Several limitations of the present review should be acknowledged when interpreting the findings summarized in this work.

First, the studies discussed involve a wide range of biological systems, including peripheral blood cells, fibroblasts, epithelial cell lines, and experimental animal models. These cellular systems differ substantially in intrinsic radiosensitivity, proliferative activity, differentiation status, and baseline transcriptional programs. As a result, differences in reported transcriptional responses may partly reflect cell-type–specific biological characteristics rather than radiation-specific effects.

Second, many experimental studies do not explicitly control for cell cycle distribution, although radiosensitivity varies considerably across cell cycle phases. Variability in the proportion of cells in G0, G1, S, or G2/M phases may therefore influence observed transcriptional responses.

Third, most transcriptomic studies focus primarily on phenotypic endpoints such as differential gene expression, while providing limited mechanistic insight into the underlying molecular pathways linking radiation exposure and biological outcomes.

Finally, a large proportion of the available evidence is derived from in vitro experiments and controlled experimental systems. While these models provide important mechanistic insights, they cannot fully reproduce the complexity of in vivo biological responses and therefore should be interpreted with caution when extrapolating to human populations.

## 8. Conclusions

Despite the widespread adoption of next-generation sequencing technologies, DNA microarrays continue to play a significant role in radiation genomics, particularly in studies addressing the biological effects of low-dose ionizing radiation. Their high degree of standardization, reproducibility, and the availability of extensive archival datasets make microarrays a methodologically justified tool for retrospective cohort studies and for the analysis of long-term molecular effects, especially in situations where repeated collection of biological material is not feasible.

As illustrated within the proposed conceptual framework (Figure 4), the interpretability and translational value of microarray-derived data are determined less by the platform itself than by the conditions of its application, including research context, data quality, analytical strategy, and appropriate biological interpretation. In this regard, inherent limitations of microarray technology, such as fixed probe design and moderate sensitivity, are effectively offset by the capacity for cross-cohort comparisons and reproducible analyses.

The integration of microarray data with multi-omics approaches and artificial intelligence methods represents a promising direction for future development, provided that such techniques are applied within interpretable and biologically grounded analytical frameworks. The supportive use of machine learning algorithms may facilitate the identification of stable radiation-induced molecular signatures and enhance molecular monitoring efforts without replacing biological interpretation with purely algorithmic models.

In summary, DNA microarrays should not be viewed as an obsolete technology, but rather as a mature methodological platform that complements NGS-based approaches. By linking historical transcriptomic datasets with contemporary multi-omics strategies, microarrays can contribute meaningfully to the advancement of long-term biomonitoring programs and the development of personalized radiation medicine.

## Figures and Tables

**Table 1:** Landscape and limitations of human transcriptomic evidence relevant to low-dose ionizing radiation (with emphasis on microarray-based studies).

Evidence Category	Representative Study/Dataset (Checked)	Human Material	Exposure Context/Dose Metric	Transcriptomic Approach	What It Actually Shows	Key Limitations (Why Low-Dose Inference is Hard)
Occupational chronic low-dose (primary human study)	Morandi et al., 2009, Radiation Research (PMID: 19772471; DOI: 10.1667/RR1545.1) (PubMed) [37]	Peripheral blood cells (workers vs. controls)	Chronic occupational; mean cumulative effective dose ~19 mSv (reported in paper) (PubMed)	Oligo-microarray	Detects sets of modulated genes in chronically exposed professionals	Small *n*; confounding (job, lifestyle, inflammation); heterogeneous cumulative dose; signal overlaps with generic stress/immune responses
Occupational low-dose (primary human study)	Fachin et al., 2009 “Gene expression profiles in radiation workers…” (PMID: 19218781) (PubMed) [38]	Lymphocytes from radiation workers	Occupational low-dose (dosimetry records)	cDNA microarray	Reports differentially expressed genes in exposed workers	Old platforms; limited replication; exposure & confounders; batch/platform legacy issues
Medical imaging, in vivo low-dose (human patients)	Kaatsch et al., 2020, Health Physics (PMID: 32167501) (PubMed) [39]	Peripheral blood	CT exposure; clinical dose context	RNA sequencing (not microarray, but human in vivo transcriptomics) (PubMed)	Shows modest, individual-dependent transcript changes; highlights timing effects	Small sample; strong inter-individual variability; timing after scan critical; not microarray (but important “reality check”)
Ex vivo human blood irradiation (benchmark for biodosimetry; microarray)	Knops et al., 2012 (PMID: 22954392) (PubMed) [40]	Human peripheral blood lymphocytes	Controlled ex vivo irradiation; includes low-dose range	Gene expression profiling (microarray-based) (PubMed)	Defines dose/time response; candidate genes for low-dose estimation	Ex vivo ≠ in vivo; dose rate differs; limited generalizability to chronic/heterogeneous exposures
Public microarray datasets enabling re-analysis	GSE23515 (GEO): blood from 24 donors, doses 0/0.1/0.5/2 Gy (https://www.ncbi.nlm.nih.gov/)	Ex vivo human blood	Controlled irradiation; includes 0.1 Gy	Microarray (public dataset)	Provides reproducible training/validation material for methods	Not ≤ 100 mSv (0.1 Gy ≈ 100 mGy); ex vivo; different labs/pipelines introduce batch effects
Integrative re-analysis/meta-modeling (NOT primary exposure study)	Liu et al., 2023 (PMCID available; uses GEO datasets incl. GSE8917/GSE43151/GSE23515) (PMC) [41]	Public human transcriptome data	Mixed designs/doses; depends on source datasets	Re-analysis/modeling	Shows what can/can’t be extracted from archived microarray data	“Second-hand evidence”: inherits all biases of original studies; mixed dose metrics; heterogeneity dominates
Mechanistic/conceptual synthesis specifically about low-dose gene expression	Sokolov & Neumann, IJMS (DOI: 10.3390/ijms17010055) (MDPI) [3]	-	-	Review/conceptual	Frames why low-dose effects are subtle & context-dependent	Not data; should not be presented as “microarray human study.”

**Table 2:** Comparison of key characteristics of microarray and NGS technologies in the context of radiation genomics.

Criterion	Microarrays	NGS (RNA-seq, WGS)
Analytical principle	Hybridization to predefined probes	Sequencing of individual DNA/RNA fragments
Ability to detect novel transcripts	Limited: analysis restricted to known genes	High: detection of novel transcripts, mutations, and alternative splicing
Sensitivity	Moderate, dependent on array density	High, enabling detection of low-abundance transcripts
Inter-platform reproducibility	Relatively high in standardized retrospective analyses (e.g., Affymetrix-Illumina)	Moderate; dependent on protocols and sequencing depth
Data archiving and re-annotation	Very high: thousands of datasets available and amenable to re-annotation	Limited: historical sequencing datasets are less standardized
Cost of analysis	Low, especially in large cohort studies	High, increasing proportionally with sequencing depth
Time required for analysis	Short	Longer; data processing and bioinformatic pipelines may take several days
Data volume	Moderate, easily manageable	Very large (gigabytes per sample), requiring substantial infrastructure
Applicability in retrospective studies	Optimal, particularly for older cohorts	Limited due to the lack of historical NGS data
Integration with AI	Feasible following cross-platform normalization and re-annotation	More direct; data are widely used in ML/AI pipelines

**Table 3:** Interpretability and reproducibility checklist for microarray-based low-dose radiation studies.

Domain	Key Interpretability Criteria	Common Limitations
Exposure assessment	Individual or cohort-specific dose reconstruction; dose metric explicitly justified (mGy/mSv)	Use of proxy exposure indicators; mixed or undefined dose units
Biospecimen and timing	Biospecimen type and collection time relative to exposure clearly reported	Latency period ignored; heterogeneous sampling windows
Platform and preprocessing	Microarray platform specified; normalization and batch correction described	Platform heterogeneity; unaddressed batch effects
Statistical robustness	Multiple testing correction applied; effect sizes reported	Reliance on nominal *p*-values; underpowered analyses
Biological specificity	Pathway-level coherence; consistency with radiation-related processes	Dominance of generic stress or inflammatory signatures
Validation strategy	Independent cohort, technical replication, or orthogonal validation (e.g., qPCR)	Absence of validation
Data transparency	Raw or processed data publicly available (e.g., GEO, ArrayExpress)	Restricted or unavailable datasets
Translational relevance	Relevance to human exposure scenarios and dose ranges explicitly discussed	Overgeneralization from in vitro or high-dose models

## Data Availability

No new data were created or analyzed in this study. Data sharing is not applicable to this article.

## Abbreviations

The following abbreviations are used in this manuscript:

AI	artificial intelligence
CNV	copy number variation(s)
DNA	deoxyribonucleic acid
GEO	Gene Expression Omnibus
MAPK	mitogen-activated protein kinase
NGS	next-generation sequencing
QC	quality control
RNA-seq	RNA sequencing
SNP	single nucleotide polymorphism(s)
SVM	support vector machine
TGF-β	transforming growth factor beta
WGS	whole-genome sequencing
